# A Systematic Review and Case Illustrations of Misdiagnosing Intracranial Aneurysms

**DOI:** 10.7759/cureus.59185

**Published:** 2024-04-28

**Authors:** Anton Konovalov, Vadim Gadzhiagaev, Anton Artemyev, Dmitry Okishev, Yuri Pilipenko, Fyodor Grebenev, Shalva Eliava

**Affiliations:** 1 Cerebrovascular Surgery, National Medical Research Center of Neurosurgery Named After N. N. Burdenko, Moscow, RUS; 2 Neurosurgery, National Medical Research Center of Neurosurgery Named After N. N. Burdenko, Moscow, RUS; 3 Neurosurgery, Moscow Regional Clinical Research Institute Named After M. F. Vladimirsky, Moscow, RUS; 4 Vascular Surgery, National Medical Research Center of Neurosurgery Named After N. N. Burdenko, Moscow, RUS

**Keywords:** clipping, intracranial aneurysm, masquerading, mimicking, false-positive diagnosis

## Abstract

Modern neuroimaging methods do not completely rule out false diagnoses of intracranial aneurysms which can lead to an unwarranted operation associated with risks of complications. However, surgical interventions for falsely diagnosed aneurysms are quite rare. The purpose of this study is to demonstrate two clinical cases of false-positive aneurysms and a systematic review of the literature dedicated to the incidence and etiology of false-positive aneurysms, identifying risk factors associated with false-positive aneurysms. A literature search in two databases (PubMed and Web of Science) using keywords "mimicking an intracranial aneurysm", "presenting as an intracranial aneurysm", "false positive intracranial aneurysms", and "neurosurgery” was conducted. A total of 243 papers were found in the initial search in two databases. Sixteen papers (including 20 patients) were included in the final analysis. There were 10 women and 10 men. The most common location of false-positive aneurysms was the bifurcation of the middle cerebral artery (MCA). In the posterior circulation, false-positive aneurysms were identified either on the basilar artery, or at the vertebro-basilar junction. The main causes of false intracranial aneurysm diagnosis included artery occlusion with vascular stump formation, infundibular widening, fenestration, arterial dissection, contrast extravasation, and venous varix. In conclusion, summarizing the results of our analysis, we can say that surgical interventions for false-positive aneurysms are an underestimated problem in vascular neurosurgery. Despite extremely rare published clinical observations, the actual frequency of erroneous surgical interventions for false-positive aneurysms is unknown.

## Introduction and background

Intracranial aneurysms pose a serious threat to the health and lives of patients, as their rupture can lead to spontaneous subarachnoid hemorrhage (SAH), often resulting in fatal outcomes. Thanks to the development and widespread availability of magnetic resonance imaging (MRI) and computerized tomography (CT), the detection of aneurysms is now possible with a sensitivity and specificity reaching 90% [1-3].

Surgical treatment of aneurysms, especially unruptured aneurysms, is the most effective preventive measure against SAH, as evidenced by numerous epidemiological studies [4,5]. However, modern neuroimaging methods do not completely rule out false diagnoses of intracranial aneurysms [3]. A false diagnosis of an aneurysm can lead to an unwarranted operation associated with risks of complications [6].

The issue of false-positive aneurysms is rarely described in the literature, with clinical situations being extremely rare [7-19]. Population studies show that the frequency of surgical interventions for falsely diagnosed aneurysms maybe 0.006% [6]. It is important to note that not only aneurysms but also other pathological conditions can be the cause of spontaneous SAH. The combination of SAH with an aneurysm-like condition can erroneously serve as an indication for emergency surgical intervention [12,13].

The purpose of this study is to demonstrate two clinical cases of false-positive aneurysms and a systematic review of the literature dedicated to the epidemiology and etiology of false-positive aneurysms, identifying risk factors associated with false-positive aneurysms.

## Review

Materials and methods

We performed a retrospective analysis of two clinical cases of patients with false-positive aneurysms who underwent surgical intervention in our institute for the period from 2021 to 2023. An assessment of the clinical picture of the disease, preoperative research methods, intraoperative picture, and treatment outcome at the time of discharge was made.

A literature search was conducted in the PubMed and Web of Science databases using the keywords "mimicking an intracranial aneurysm", "mimicking an aneurysm", "presenting as an intracranial aneurysm", "presenting as an aneurysm", "false positive intracranial aneurysms", "false positive aneurysms", and "neurosurgery". We used the following search queries:

(mimicking an intracranial aneurysm)

(mimicking an aneurysm) AND (neurosurgery)

(presenting as an intracranial aneurysm)

(presenting as an aneurysm) AND (neurosurgery)

(false positive intracranial aneurysm)

(false positive aneurysm) AND (neurosurgery)

Inclusion and Exclusion Criteria

The inclusion criteria for this study involved publications written in English and providing full access to the article. Additionally, the included articles needed to contain information about the primary diagnosis and the diagnostic method used, as well as information about the results of the clinical case.

Conversely, publications duplicating information from other sources were excluded, along with publications containing only abstracts, conference theses, reviews, or article or book reviews. Articles that were not available for full-text viewing were also excluded from consideration.

**Figure 1:**
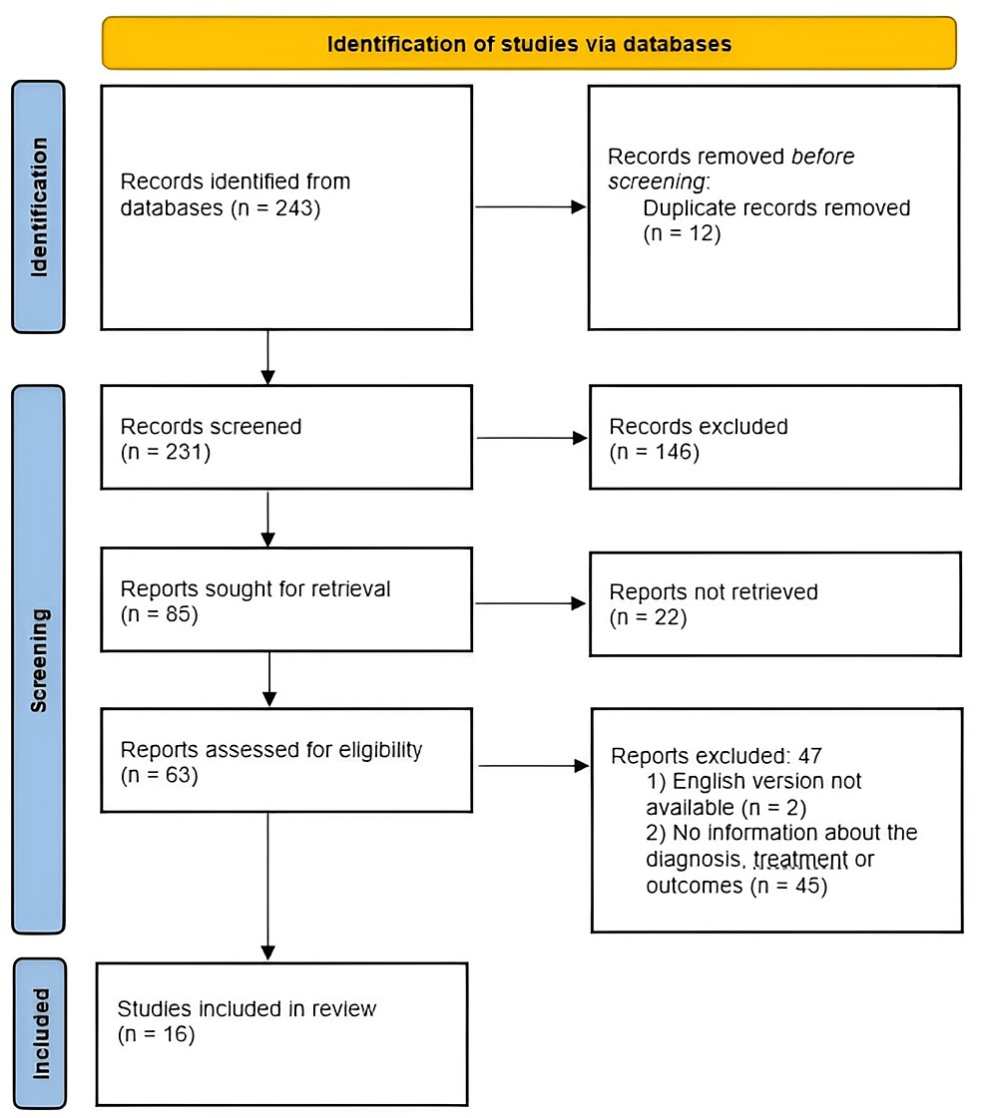
PRISMA flow chart depicting the process of study selection PRISMA: Preferred Reporting Items for Systematic Reviews and Meta-Analyses

As a result, 16 publications were selected according to the Preferred Reporting Items for Systematic Review and Meta-Analysis (PRISMA) protocol (Figure 1).

Results

Process of Study Selection

A total of 227 papers were found in the initial search in the PubMed database, and 16 were found in Web of Science (243 in total). Due to duplication, 12 papers were removed. After primary screening, 168 papers were removed due to noncompliance with the inclusion criteria. After studying the full texts of the articles, 54 papers were excluded. Sixteen papers were included in the final analysis.

General Characteristics of the Group

This review analyzed 20 patients from 16 studies. The median age was 53.5 years (range 15-74). Among the patients, there were 10 women and 10 men.

Distribution of False-Positive Aneurysms by Location

The majority of false-positive aneurysm cases were related to the arteries of the anterior circulation (85.0%) (Table 1). The most common location was the bifurcation of the middle cerebral artery (MCA) (40.0% of all cases). There were no false-positive aneurysms of the M1 segment or distal segments of the MCA in the studies reviewed.

**Table 1:** Location of false-positive aneurysms and corresponding underlying structures mimicking aneurysms according to the studies SAH: spontaneous subarachnoid hemorrhage; ACA: anterior cerebral artery; AComA: anterior communicating artery; ICA: internal carotid artery; MCA: middle cerebral artery; VBJ: vertebrobasilar junction

Underlying structure	Total number of cases	By location
Vascular stump due to branch occlusion	11	MCA bifurcation (7), A1 ACA segment (2), supraclinoid ICA (1), VBJ (1)
Infundibular widening of the origin of the small vessel	4	Supraclinoid ICA (2), AComA perforator (2)
Arterial fenestration	1	AComA (1)
Arterial wall dissection with subsequent extraluminal thrombus formation	1	Supraclinoid ICA (1)
Near-artery contrast extravasation in patients with SAH	2	Basilar artery (2)
Venous vessel (or venous varix) traversing the common aneurysm formation site	1	MCA bifurcation (1)

False-positive aneurysms of the supraclinoid segment of the internal carotid artery (ICA) were slightly less common (20.0%). There were no false-positive aneurysms noted in the ophthalmic segment or at the bifurcation of the ICA.

In the anterior cerebral artery (ACA), false-positive aneurysms were located either in the A1 segment (10.0%) or in the region of the anterior communicating artery (ACoA) complex (15.0%). There were no false-positive aneurysms of the distal segments of the ACA.

In the posterior circulation, false-positive aneurysms were identified either on the trunk of the basilar artery (BA) (10.0%) or at the vertebro-basilar junction (VBJ) (5.0%). False-positive aneurysms of the BA bifurcation, posterior cerebral artery (PCA), superior cerebellar artery (SCA), anterior inferior cerebellar artery (AICA), and posterior inferior cerebellar artery (PICA) were not mentioned in the studies.

Artery Occlusion With Vascular Stump Formation as a Cause of False Aneurysm Diagnosis

This scenario is the most common cause of false intracranial aneurysm diagnosis. Most often, this variant was detected at the MCA bifurcation (63.6%) at the origin of one of the M2 branches, less frequently - in the A1 segment area of the ACA (18.1%), at the VBJ (9.1%), or at the origin of the posterior communicating artery (PCoA) on the supraclinoid segment of the ICA (9.1%).

In a large number of cases (36.7%), the cause of artery occlusion was progressive atherosclerotic changes in its wall followed by obliteration. This variant was observed exclusively in the MCA area: in two cases - in the trifurcation area, in one case - in the bifurcation area, and in one case - in the trifurcation area of a secondary branch. In two instances, false-positive aneurysms were incidental findings, while in two others, there were clinical signs of cerebral ischemia: one patient was admitted with an acute cerebral infarction, and the other had recurring transient ischemic attacks (TIAs). In three cases (including the patient with TIA), MRI showed no signs of brain ischemia, and two of them showed signs of the moyamoya phenomenon. The patient with acute cerebral infarction also suffered from systemic lupus erythematosus.

Artery occlusion due to dissection was equally common (36.7%) among the patients studied. It most often developed in the MCA bifurcation area (three cases) and less frequently at the VBJ (one case). In all these cases, the patients had verified SAH, and in one of them, artery occlusion (one of the M2 branches) led to acute cerebral infarction.

In three cases (27.3%), occlusion occurred in the area of one of the A1 segments of the ACA (two cases) and the origin of the PCoA (one case). All patients in this subgroup were elderly (62-70 years old). The most likely cause of occlusion was progressive stenosis against the background of the original congenital hypoplasia of the vessel (A1 segment or PCoA). In one of these patients (suspected PCoA aneurysm), the reason for the examination was a sudden headache; however, neither CT nor microsurgical revision revealed any SAH. In another case, SAH was detected on CT, but the source of the hemorrhage could not be found during surgery; thus, a non-aneurysmal non-perimesencephalic SAH was verified. In the last case, the patient showed symptoms of chronic cerebral ischemia due to ICA occlusion. Preoperative angiography revealed a moyamoya phenomenon. During open surgery, no aneurysm was found in the ACoA and A1 segment area, and encephaloduroarteriosynangiosis (EDAS) was performed to treat chronic ischemia.

Infundibular Widening of the Artery Orifice as a Cause of False Aneurysm Diagnosis

This variant was described in four cases (20.0%). In two cases, an enlargement of the orifice of a perforating branch of the ACoA complex was described, and in two cases, of one of the trunks of the anterior choroidal artery (AChA).

One of the patients was examined due to a sudden severe headache caused by intracerebral hemorrhage (ICH). No signs of SAH were found. CT angiography (CTA) suspected the presence of an ACoA aneurysm, which was thought to be the cause of the ICH. However, during the operation, it was found that the structure considered to be an aneurysm was actually the infundibular widening of one of the trunks of the recurrent artery of Heubner, originating from the ACoA. There were no signs of SAH, and this dilation was not connected to the cavity of the intracerebral hematoma.

The second patient had two ACoA aneurysms detected incidentally. However, during surgery, it was discovered that one of the aneurysms was actually the dilation of the orifice of a perforating branch of the ACoA. Clipping was performed on the remaining aneurysm, while the dilated orifice was left under observation.

In the two remaining patients who were examined for minor headaches, the erroneously diagnosed aneurysms turned out to be dilated orifices of one of the AChA trunks. In one case, only an open revision was performed, while in the second, a part of the dilation was clipped due to significant thinning of its walls and the presence of diverticula.

Artery Fenestration Associated With the Diagnosis of False Aneurysms

This variant was identified in only one patient. Initially, the bulge was small. The reason for the open surgery in this patient was the growth of the bulge over time (over two years) - from 4.1 mm to 5 mm. During the operation, it was found that this bulge was an enlargement of one part of the fenestrated ACoA. Preoperative angiography also revealed a small MCA aneurysm, but revision of this area during surgery was not performed, and the patient was left under observation.

Subadventitial Thrombus Mimicking an Intracranial Aneurysm

A 15-year-old patient underwent surgery for temporal ganglioglioma and structural epilepsy. Postoperative CT and CTA revealed a hyperdense focus in the MCA area. The formation of a pseudoaneurysm due to dissection and hemorrhage risks was suspected, prompting a repeat operation. However, intraoperatively, it was discovered that this focus was a blood clot resulting from slow, nonintensive subadventitial bleeding from a small vessel adjacent to the arterial wall. After clot removal and coagulation of the vasa vasorum, no defects were found in the MCA wall.

Contrast Extravasation Mimicking an Intracranial Aneurysm

This type of false-positive aneurysm was described in two patients with SAH. CTA performed within the first day after SAH identified signs of an aneurysm at the BA apex. However, on digital subtraction angiography (DSA) and follow-up CTA six days later, there were no signs of an aneurysm. The cause of the SAH could not be determined. Thus, the phenomenon detected on CTA was interpreted as contrast extravasation from the hemorrhage source.

Venous Varices Mimicking Intracranial Aneurysms

The last patient, examined due to severe headaches, was found to have an ICH without SAH. CTA indicated signs of an aneurysm at the MCA bifurcation. However, the patient's clinical picture over an extended period showed chemosis, and MRI identified an enlargement of the superior ophthalmic vein, which steered the diagnosis away from a ruptured aneurysm. DSA was performed, revealing a dural arteriovenous fistula (dAVF) with drainage into the transverse and sigmoid sinuses and partially into the SOV through a venous varix. Open removal of the hematoma and disconnection of the venous drainage were carried out.

Treatment Types Utilized in Patients With False-Positive Aneurysms

Fourteen patients (70.0%) underwent microsurgical procedures. For 13 of them, the primary preoperative diagnosis was an intracranial aneurysm, and surgeons were convinced of the need for clipping. The remaining patient had the aneurysm diagnosis excluded, but an open surgery was still conducted to remove a hematoma and perform an open venous disconnection of the dAVF. No treatment-related complications were noted in the operated patients.

Six patients (30.0%) were treated conservatively, five of whom were treated for vasospasm due to SAH, including endovascular chemoangioplasty. The remaining patient was simply under dynamic observation.

Illustrative cases

Case 1

**Figure 2:**
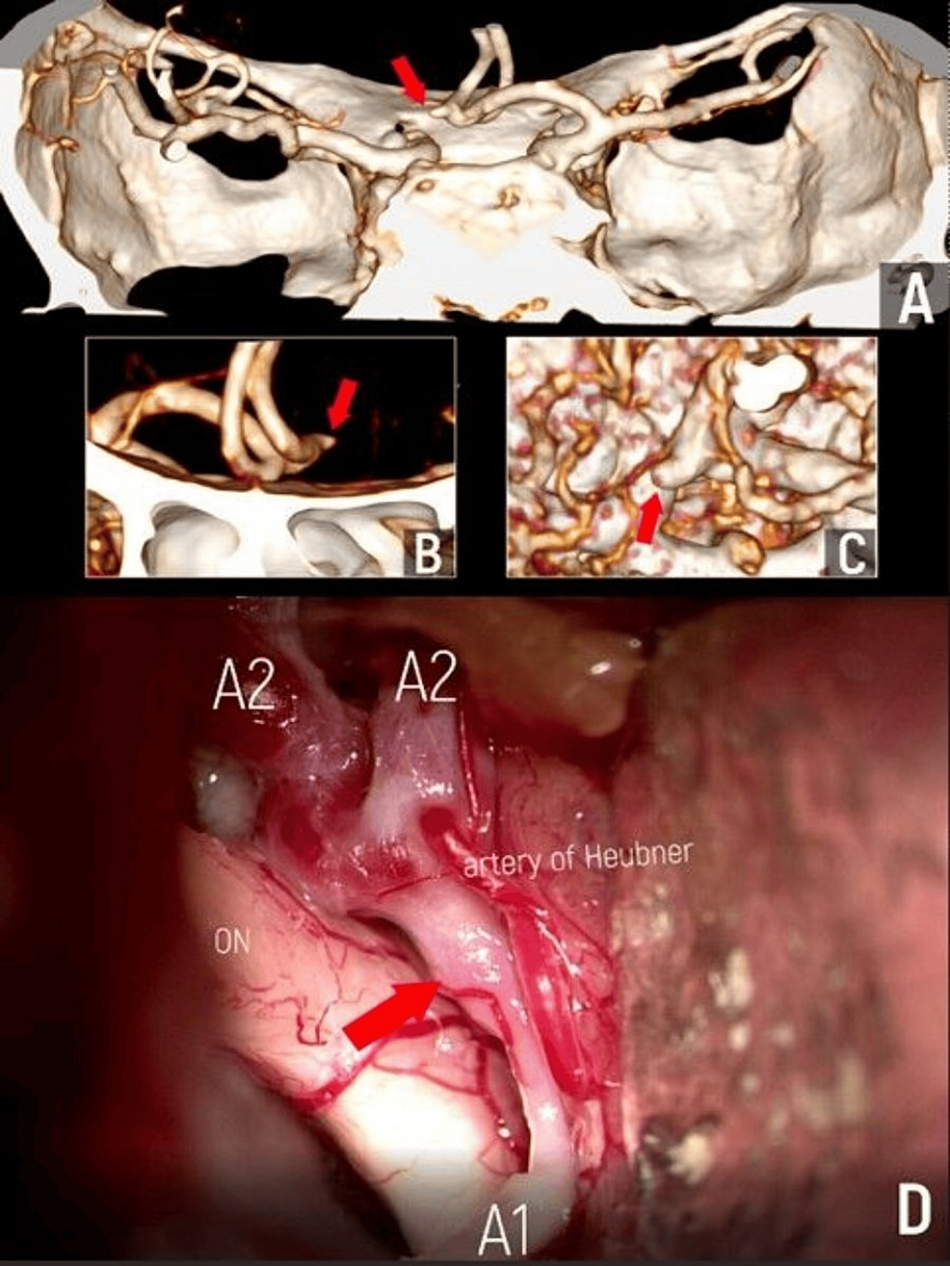
Illustrative Case 1 A, B, C - different projections on preoperative 3D CTA, aplasia of the left A1 segment had been suspected; D - intraoperative view of the dilated distal part of the left A1 segment (red arrow) mimicking an aneurysm, both A2 segments and the left recurrent branch of Heubner are visualized, as well. CTA: computed tomography angiography The figure was created by the authors.

The patient, a 44-year-old woman, had been experiencing migraine-type headaches for a year. A neurologist recommended an MRI of the head, which revealed signs of an aneurysm of the ACoA. After consulting with a neurosurgeon, she was referred for CTA (Figures 2A-2C). 3D reconstruction mode revealed a cone-shaped aneurysm at the angle of the ACA - A2 on the left, with signs of hypoplasia of the left A1. Considering her young age, the presence of a cone-shaped aneurysm, and the risk of intracranial hemorrhage, the patient was recommended to undergo microsurgical aneurysm clipping. The operation was performed as scheduled. Intraoperatively, signs of obliteration of the A1 segment on the left were found, with signs of a functioning distal part of A1 on the left mimicking an aneurysm (Figure 2D). Clipping of the functioning vessel segment was performed with a straight titanium clip. The postoperative period was satisfactory. The patient was informed about the anatomical features mimicking the aneurysm and the surgical treatment tactics chosen by the surgeon intraoperatively. She was discharged on the seventh day after the operation without complications.

Case 2

**Figure 3:**
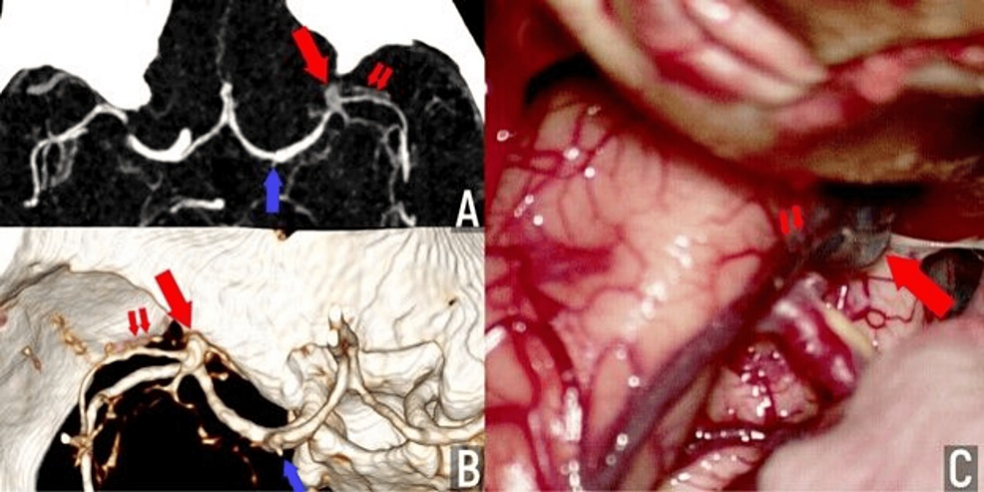
Illustrative Case 2 А, В - axial CTA (A) and 3D CTA (B) in a mixed arterio-venous phase depicting two aneurysms: tiny left A1 segment aneurysm (blue arrow) and small MCA bifurcation aneurysm (big red arrow), a venous branch (small red arrows) traversing the aneurysm site is recognizable as well; С - intraoperative view showing dilated vein (small red arrows) entering the spheno-parietal sinus (big red arrow), lying close to the MCA bifurcation, no aneurysms were found at this site, only A1 segment aneurysm was clipped. MCA: middle cerebral artery; CTA: computed tomography angiography The figure was created by the authors.

The patient, a 54-year-old man, had been suffering from hypertension for several years. He was examined due to an attack of severe headaches and a rise in blood pressure to 180 mmHg. MRI and CTA revealed signs of two aneurysms: at the bifurcation of the left MCA and a miliary aneurysm at the initial sections of the A1 segment on the left (Figures 3A-3B). Considering the risks of intracranial hemorrhage, microsurgical treatment was suggested to clip both aneurysms. Given the confidence in the aneurysm diagnosis and the invasiveness of the examination, direct cerebral angiography was not performed. The patient was prepared for a scheduled microsurgical operation to clip the aneurysms. The first step involved access to the basal cisterns, identification, and clipping of the aneurysm of the A1 segment on the left. In the second step, during trans-silvan dissection and revision of the M1 segment bifurcation area on the left, no signs of an aneurysm were found (Figure 3C). A venous varix of the sphenoparietal sinus adjacent to the bifurcation area of the left MCA was found, mimicking a cerebral aneurysm on CTA in the mixed arteriovenous phase. The postoperative period was satisfactory; the patient was discharged on the eighth day after the operation without complications.

Discussion

All the studies we analyzed were case reports or small series of observations, not exceeding three clinical cases. None of the studies provided information on how frequently such situations occur. There are no data on the frequency of false-positive aneurysms, both actual and presumed. It seems that such a problem does not exist, and the published series are extremely rare observations. However, with the development of MRI and CT diagnostics, increased accessibility, and various screening programs, for example, for neurovascular diseases, the frequency of false-positive diagnoses may increase [3,6]. False-positive aneurysms are a serious problem, especially in the case of population screening. The frequency of false-positive results varies depending on the type of study used and the experience of the specialist [20]. Magnetic resonance angiography (MRA) has a higher frequency of false-positive results than CTA [21]. Several studies have reported the frequency of false-positive results related to aneurysm screening. Okuyama et al. reported that three out of 66 (4.5%) aneurysms detected by MRA or CTA were false positive [22]. A meta-analysis conducted by Hiratsuka et al. showed that the overall specificity for diagnosing an aneurysm via MRA ranges from 50% to 100% [23]. In this case, the range of radiological false-positive diagnoses can exceed 5%, and the true frequency of false-positive aneurysms remains unknown.

Diagnosis of False-Positive Aneurysms

For the diagnosis of cerebral aneurysms, MRA or CTA are typically used [1,8,21]. Aneurysms less than 5 mm are difficult to detect in the initial images, so it is recommended to use 2D and 3D postprocessing. It is also recommended to view the original data with wide window settings to ensure differentiation between arteries, veins, areas filled with contrast material, bone, and calcifications.

However, image assessment should begin with the examination of the original images. This is because even the most advanced 3D imaging methods can lead to the loss of important diagnostic information. Partial thrombosis, dissection, and calcinosis may be missed or misinterpreted if the original images are not reviewed.

The cause of a false-positive result can be the inaccuracy of performing CTA, resulting in a mixed-phase scan. To obtain a "purer" arterial phase, a high contrast injection rate is needed, no less than 4 mL/s with minimal delay. Scanning should be conducted in the caudo-cranial direction. Additionally, when performing MR angiography, venous structures do not appear in the images, which can be used for differential diagnosis in doubtful cases. In situations where there are doubts about the presence of a saccular aneurysm in both CTA and MRA, an invasive DSA study may be recommended [21]. DSA, especially in 3D mode, is the gold standard for confirming the diagnosis but cannot be used as a screening method. DSA is associated with a risk of complications: neurological in 0.1-0.5% of cases and local surgical complications related to the puncture of a large artery in 1-4% [24]. Moreover, in the situation with a functioning vessel orifice against the background of distal occlusion (for example, occlusion of M2 or A1 segments), it is extremely difficult to make the correct diagnosis with DSA [13,19,25]. The appearance of a vascular stump on CTA is extremely difficult to distinguish from a true aneurysm. As mentioned earlier, this scenario is the most common in false aneurysm diagnostics. Most often, the arterial stump, mimicking an aneurysm, was identified in the area of the division of the main MCA trunk. In some cases, patients exhibited trifurcation of the MCA, and the occlusion of one of the secondary branches with the formation of a stump created a typical picture of MCA bifurcation and a corresponding aneurysm in a typical location. Thus, the presence of only one of the M2 branches (in the case of MCA bifurcation) and a structure resembling an aneurysm should raise suspicion of occlusion of the second branch with the formation of a stump. For an unprepared doctor, such similar manifestations can lead to unjustified surgical intervention: microsurgical clipping or endovascular treatment. There are no reliable methods to distinguish these two conditions. There is experience in using MRI in SPACE mode, demonstrating the presence of a thrombosed or obliterated vessel along its length [8].

Cases of arterial dissection, leading first to SAH and then to occlusion, pose a particular complexity [13]. In this case, a picture of an aneurysmal SAH develops, and due to the doctors' conviction about the need for emergency exclusion of the aneurysm, the possibility of a false-positive aneurysm is dismissed. Early development of ischemic changes in the brain (before the peak of vasospasm) due to acute artery occlusion, the nonmassive nature of the SAH, should alert surgeons to the possibility of dissection. Such a clinical picture may occur in approximately 3% of SAH cases [8].

The literature thoroughly describes the problem of infundibular widenings of the PCoA origin [26]. In most cases, they require treatment. However, in cases of a more distal location (in the area of the AChA origin), there may be suspicions of an intracranial aneurysm. As the literature data show, such findings can be an expansion of one of the AChA trunks in cases where it has a diffuse structure [11]. Clipping such dilations can lead to severe complications, such as infarction in the area of the internal capsule. Additionally, as our analysis shows, such dilations can occur on the ACoA - at the origins of the perforating branches [7]. There is no consensus in the literature on the treatment tactics for such dilations. Nevertheless, with small dilation sizes, we recommend observation to avoid gross mnemonic and cognitive disorders after surgery.

Fenestration of the arteries itself increases the risk of developing an intracranial aneurysm. However, in some cases, with dilation or occlusion of part of the fenestrated artery, an intracranial aneurysm may be misdiagnosed [10,16,27]. In one of the cases studied in this review, occlusion of the proximal part of the fenestrated vertebral artery occurred against the background of dissection with SAH, creating a picture of a VBJ aneurysm [16].

It should be remembered that on CTA, performed in the first 24 hours after SAH, aneurysm-like areas of contrast extravasation may occur [12]. In the case of an atypical radiological picture and a high probability of the absence of an aneurysm, it is necessary to conduct DSA and repeat CTA angiography after a while.

Additionally, one of the types of false-positive aneurysms is venous varices. They most often form under conditions of arteriovenous shunting (with AVMs and dAVFs) [9]. To prevent misdiagnosis, it is also necessary to take into account other manifestations of arteriovenous shunting (chemosis, pulsating noise, radiological signs).

Unjustified Surgical Interventions

In none of the studies we presented, we found data on how often such situations occur in practice. There is also no single opinion on how to regard these surgical interventions as a diagnostic intervention or a mistake. The diagnosis of an intracranial aneurysm leads to psychological problems, including anxiety, stress, and depression [28,29]. In some cases, after a radiological diagnosis is established, patients limit themselves in physical activity, work, hobbies, sex, etc. An incorrect diagnosis implies potential harm, both physical and psychological, for patients and the risk of surgical complications without apparent benefits.

This issue requires attention and identification of risk factors for false-positive aneurysms. These, in our view, may include CTA in mixed arterial and venous phases, the presence of falx petrifications in the A3 segment area, ischemic lesions in cases of small ACA and MCA aneurysms with an elongated "base", and the presence of fenestration. All these situations require accurate diagnosis and confirmation of the diagnosis before surgical intervention. Nevertheless, we understand that with SAH and the presence of suspicion of an aneurysm, delaying the diagnostic process before surgical treatment can have negative consequences.

Doctors may feel anxiety due to incorrect diagnoses and fear of legal liability. This issue has become more significant for radiologists, who are now more frequently confronted with the risks of medical-legal prosecution. Medical liability is a serious concern for high-risk specialties such as neuroradiology and neurosurgery, where an incorrect diagnosis can lead to extremely adverse consequences. This may be a reason for the lack of data and discussions about the frequency of false-positive aneurysms and surgical interventions in the literature. In our opinion, it is impossible to eliminate instances of unwarranted surgical interventions in cases of false-positive aneurysms.

## Conclusions

Surgical interventions for false-positive aneurysms are an underestimated problem in vascular neurosurgery. Modern diagnostic methods significantly reduce the frequency of false-positive aneurysms but do not guarantee their complete absence. Despite extremely rarely published clinical observations, the actual frequency of erroneous surgical interventions for false-positive aneurysms is unknown. Further research is needed to establish the epidemiology and factors of incorrect diagnosis and treatment of false-positive aneurysms.

## References

[1] White PM, Wardlaw JM, Easton V (2000). Can noninvasive imaging accurately depict intracranial aneurysms? A systematic review. Radiology.

[2] Rustemi O, Alaraj A, Shakur SF (2015). Detection of unruptured intracranial aneurysms on noninvasive imaging. Is there still a role for digital subtraction angiography?. Surg Neurol Int.

[3] Mine B, Pezzullo M, Roque G, David P, Metens T, Lubicz B (2015). Detection and characterization of unruptured intracranial aneurysms: comparison of 3T MRA and DSA. J Neuroradiol.

[4] Van Hoe W, van Loon J, Demeestere J, Lemmens R, Peluso J, De Vleeschouwer S (2021). Screening for intracranial aneurysms in individuals with a positive first-degree family history: a systematic review. World Neurosurg.

[5] Ikawa F, Morita A, Nakayama T (2020). A register-based SAH study in Japan: high incidence rate and recent decline trend based on lifestyle. J Neurosurg.

[6] Konovalov A, Grebenev F, Savinkov R (2023). Mathematical analysis of the effectiveness of screening for intracranial aneurysms in first-degree relatives of persons with subarachnoid hemorrhage. World Neurosurg.

[7] Park J, Kang DH (2013). Infundibular widening mimicking anterior communicating artery aneurysm: report of 2 cases. J Neurosurg.

[8] Khattar NK, White AC, Adams SW, Aljuboori ZS, Wilder MJ, Downs RK, James RF (2019). Republished: MRI SPACE sequence confirmation of occluded MCA M2 dissection stump masquerading as a ruptured MCA aneurysm. J Neurointerv Surg.

[9] Onuma K, Yanaka K, Tsukada A, Nakamura K, Matsumaru Y, Ishikawa E (2022). Intracranial varix of the transverse-sigmoid dural arteriovenous fistula mimicking a ruptured middle cerebral artery aneurysm: a case report. Surg Neurol Int.

[10] Tsukada A, Yanaka K, Takeda H, Onuma K, Takada M, Nakamura K, Ishikawa E (2023). Fenestrated anterior communicating artery complex mimicking an unruptured aneurysm: diagnostic pitfall. Asian J Neurosurg.

[11] Park J, Kim JS (2023). Infundibular widening of angiographically invisible duplicate anterior choroidal artery mimicking typical anterior choroidal artery aneurysm. J Korean Neurosurg Soc.

[12] Stetson ND, Pile-Spellman J, Brisman JL (2012). Contrast extravasation on computed tomographic angiography mimicking a basilar artery aneurysm in angiogram-negative subarachnoid hemorrhage: report of 2 cases. Neurosurgery.

[13] Lee JM (2022). Subarachnoid hemorrhage due to middle cerebral artery dissection mimicking aneurysm - case report. Radiol Case Rep.

[14] Uneda A, Yabuno S, Kanda T, Suzuki K, Hirashita K, Yunoki M, Yoshino K (2017). Cavernous angioma presenting with subarachnoid hemorrhage which was diffusely distributed in the basal cisterns and mimicked intracranial aneurysm rupture. Surg Neurol Int.

[15] Bharatha A, Fox AJ, Aviv RI, Symons SP (2007). CT angiographic depiction of a supraclinoid ICA fenestration mimicking aneurysm, confirmed with catheter angiography. Surg Radiol Anat.

[16] Kalia KK, Pollack IF, Yonas H (1992). A partially thrombosed, fenestrated basilar artery mimicking an aneurysm of the vertebrobasilar junction: case report. Neurosurgery.

[17] Kawanishi M, Sakaguchi I, Miyake H (2003). Occlusion of the posterior communicating artery mimicking cerebral aneurysm: case report. Neurol Res.

[18] Liu Y, Guo G, Lin Z, Zhao L, Hernesniemi J, Li C, Andrade-Barazarte H (2022). Occlusion of the anterior cerebral artery mimicking a cerebral aneurysm: clinical presentation and literature review. J Neurol Surg A Cent Eur Neurosurg.

[19] Takeuchi S, Nawashiro H, Otani N, Shima K (2015). Middle cerebral artery branch occlusion mimicking an aneurysm. Asian J Neurosurg.

[20] Jang M, Kim JH, Park JW (2020). Features of "false positive" unruptured intracranial aneurysms on screening magnetic resonance angiography. PLoS One.

[21] Cho SH, Lee JY, Ryu KH, Suh DC (2018). Diagnosis of cerebral aneurysm via magnetic resonance angiography screening: emphasis on legal responsibility increases false positive rate. Neurointervention.

[22] Okuyama T, Saito K, Hirano A, Takahashi A, Hashimoto Y, Inagaki T (1997). Diagnosis of unruptured cerebral aneurysms using magnetic resonance angiography and three dimensional computed tomographic angiography [Article in Japanese]. No Shinkei Geka.

[23] Hiratsuka Y, Miki H, Kiriyama I (2008). Diagnosis of unruptured intracranial aneurysms: 3T MR angiography versus 64-channel multi-detector row CT angiography. Magn Reson Med Sci.

[24] Kaufmann TJ, Huston J 3rd, Mandrekar JN, Schleck CD, Thielen KR, Kallmes DF (2007). Complications of diagnostic cerebral angiography: evaluation of 19,826 consecutive patients. Radiology.

[25] Yu J, Zhang Y, Wang H (2013). Occluded middle cerebral artery vascular stump mimicking aneurysm: case report and review of literature. Pak J Med Sci.

[26] Yu J, Xu B, Liu Y, Xu B, Xu K (2015). Progress in treating ruptured infundibular dilatation at the origin of the intracranial posterior communicating artery. Int J Clin Exp Med.

[27] Weil AG, Bojanowski MW, Scholtes F, Darsaut TE, Signorelli F, Weill A (2011). Angiographic pitfall: duplicated tapered A1 segment of the anterior cerebral artery mimicking an anterior communicating artery aneurysm. Interv Neuroradiol.

[28] Buijs JE, Greebe P, Rinkel GJ (2012). Quality of life, anxiety, and depression in patients with an unruptured intracranial aneurysm with or without aneurysm occlusion. Neurosurgery.

[29] Garzon-Muvdi T, Yang W, Luksik AS, Ruiz-Valls A, Tamargo RJ, Caplan J, Tamargo RJ (2017). Postoperative delayed paradoxical depression after uncomplicated unruptured intracranial aneurysm surgery. World Neurosurg.

